# Bibliometric Insights in Advances of Anaplastic Thyroid Cancer: Research Landscapes, Turning Points, and Global Trends

**DOI:** 10.3389/fonc.2021.769807

**Published:** 2021-11-24

**Authors:** Hanyu Wang, Yuxin Yu, Kang Wang, Hui Sun

**Affiliations:** ^1^ Department of Endocrinology, Union Hospital, Tongji Medical College, Huazhong University of Science and Technology, Wuhan, China; ^2^ Department of Forensic Medicine, Tongji Medical College, Huazhong University of Science and Technology, Wuhan, China; ^3^ Department of Forensic Medicine, Nanjing Medical University, Nanjing, China

**Keywords:** bibliometric, anaplastic thyroid cancer, visualized maps, quantitative analysis, research frontiers

## Abstract

**Background:**

Thyroid cancers are the most common endocrine malignancies with a dramatic increase in incidences. Anaplastic thyroid cancer is a rare but deadly form among thyroid cancers. To better understand of this field, we assessed the global scientific outputs and tried to depict its overview *via* bibliometric methods.

**Methods:**

Approximately 1,492 science publications published between 1997 and 2020 were included by systematic retrieval in the WoS database. The general information of them was characterized, and the developmental skeleton and research frontiers were explored.

**Results:**

The article number in this field has been increasing in the past 24 years. North America, East Asia, and Western Europe have reached remarkable achievements. Mutations of *BARF* and *TERT* and their downstream pathways have attracted researchers’ attention, where genetic diagnosis provides new clinical insight and several targeted therapeutic approaches have been on the clinical trial.

**Conclusions:**

Numerous efforts have been made to figure out gene expression reprogramming of anaplastic thyroid cancer and key mechanism in driving its dedifferentiation, invasion and migration process. Targeted therapy, immunotherapy, and systematic combination therapy are the recent current research hotspots. These results provide insightful clues for the funding direction and the potential breakthrough direction of the anaplastic thyroid cancer study.

## Introduction

The thyroid gland is one such endocrine organ that plays a vital role in human metabolism. As the largest gland located in front of the neck, it produces certain hormones in regulating physiological activities, but sometimes it will fail in function and become malignant (1). Thyroid cancer is a type of cancer that originates from the thyroid gland. The cells begin proliferating abnormally and inhibit the survival and work of normal cells, causing compression of the neck structures and various symptoms. Thyroid cancers are amongst the most prevalent medical conditions. Globally, 3.2 million people were affected by thyroid cancer, leading to 31,900 deaths in 2015 (2, 3). Due to the progressed understanding of thyroid and better detection methods, its global impact increases each year, causing billions of dollars burden (4–6). In 2020, thyroid cancer has ranked in ninth place for incidence and has been responsible for 586,000 cases worldwide, leading to a considerable global public health issue (7).

Thyroid cancers can be classified into four main types according to their histopathological characteristics: papillary thyroid cancer (PTC), follicular thyroid cancer (FTC), medullary thyroid cancer (MTC), and anaplastic thyroid cancer (ATC). Among them, ATC is a rare but fatal form of thyroid cancer, reaching to only 1–2% of cases but a 5-month median survival and a 20% 1-year survival rate (8, 9). It is remarkable that 40–50% of total thyroid cancer-related deaths come from ATC in the United States (8, 10). Unlike well-differentiated thyroid cancers, ATC is marked by high degree of cellular proliferation, poor differentiation, atypical mitosis, aggressive behavior, and high risk for subsequent distant metastasis (11, 12). Thus, diagnosis of ATC is often suspected in patients with large and rapidly growing neck masses, symptoms of invasion to surrounding tissues, and distant metastases to lungs, bones, and brains (13–15). However, ATC possesses poor differentiation and various histopathological spectrums, and lacks typical morphologic characteristics and specific biomarkers, which challenges the diagnosis of ATC. It has been proved that ATC possesses significant mutation accumulation and widespread genomic disruptions (i.e., *TP53*, *BRAF*, *TERT* promoter, and *PI3K/AKT* pathway, etc.), suggesting the extreme difficulty in chemotherapy and targeted therapies (10, 16–18). Meanwhile, ATC is intractable to be cured neither by surgery nor radiotherapy because of its propensity for adjacent invasion and distant metastasis (10, 16). Despite the progress in studying histopathological and genomic spectrum of ATC, it remains resistant to standard therapies and comes out the poor prognosis.

Owing to the high mortality rate and corresponding social pressure, more profound understanding of the development of ATC’s scientific landscapes is necessary. It contributes to researchers’ investigation and macroscopic grasp of the etiology and clinical treatment. Therefore, we aimed to conduct a bibliometric network analysis, which could provide an objective measurement in scientific literature and aggregate the opinions of multiple scholars in the field of ATC. Through a quantitative and qualitative analysis of the related literature, it is likely for researchers to possess insight on ATC about its developmental skeleton, current landscape, and possible future directions (19–21).

## Materials and Methods

**Figure 1:**
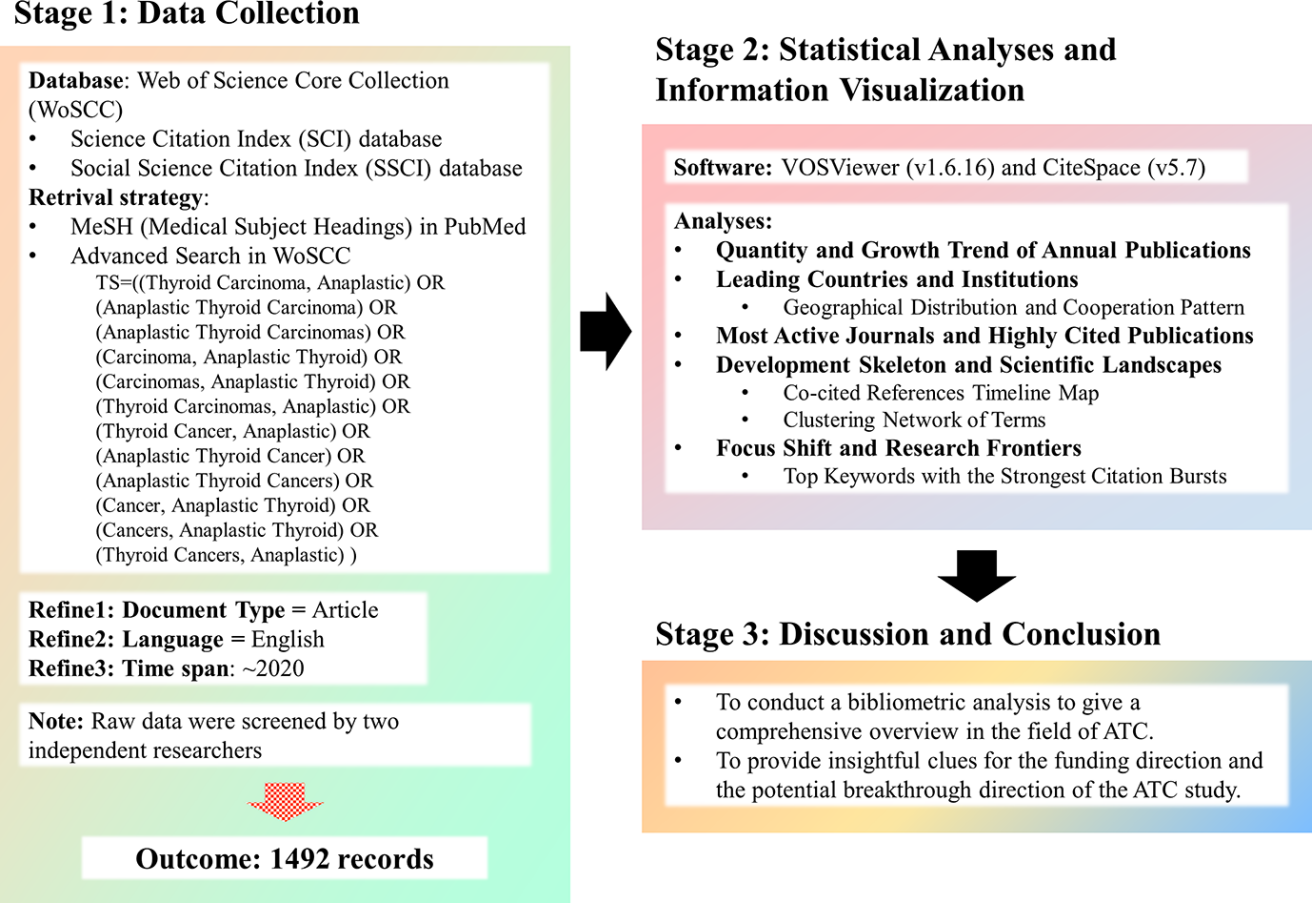
The flow chart of the methodology.

A database was built to retrieve related literature on the topic of ATC for bibliometric studies *via* the Science Citation Index (SCI) database and the Social Science Citation Index (SSCI) database from the Web of Science (WoS). For a better retrieval accuracy, topic entry items were obtained from a standardized Medical Subject Headings (MeSH) list in the National Library of Medicine. The searching strategy was designed as follows: (Topic = (‘Thyroid Carcinoma, Anaplastic’) OR (‘Anaplastic Thyroid Carcinoma’) OR (‘Anaplastic Thyroid Carcinomas’) OR (‘Carcinoma, Anaplastic Thyroid’) OR (‘Carcinomas, Anaplastic Thyroid’) OR (‘Thyroid Carcinomas, Anaplastic’) OR (‘Thyroid Cancer, Anaplastic’) OR (‘Anaplastic Thyroid Cancer’) OR (‘Anaplastic Thyroid Cancers’) OR (‘Cancer, Anaplastic Thyroid’) OR (‘Cancers, Anaplastic Thyroid’) OR (‘Thyroid Cancers, Anaplastic’)). This search strategy retrieved 2,141 records initially within English literature up to February 5, 2021. Subsequently, to minimize bias in our analysis as much as possible, “original article” was set as the inclusion criteria. The raw records were downloaded and filtered by two independent investigators from different affiliations to reject irrelevant studies. Finally, the four records published in 2021 were excluded, and 1,492 literatures were obtained for in-depth analysis (**Figure 1**).

Bibliometric tools VOSViewer 1.6.16 (22, 23) and CiteSpace 5.7 (24–26) were chosen to make statistical analysis and convert the raw data into visualization. The number of annual publications in each category and the pattern of contribution and collaboration among countries or districts were visualized. Reference co-citation analysis was performed to outline the development skeleton. Clustered terms extracted from the title and abstract were adopted for depicting research landscapes. Detected burst keywords were utilized for forecasting the possible hotspots and research frontiers in the future.

## Results

### Growth and Category of Annual Publications

**Figure 2:**
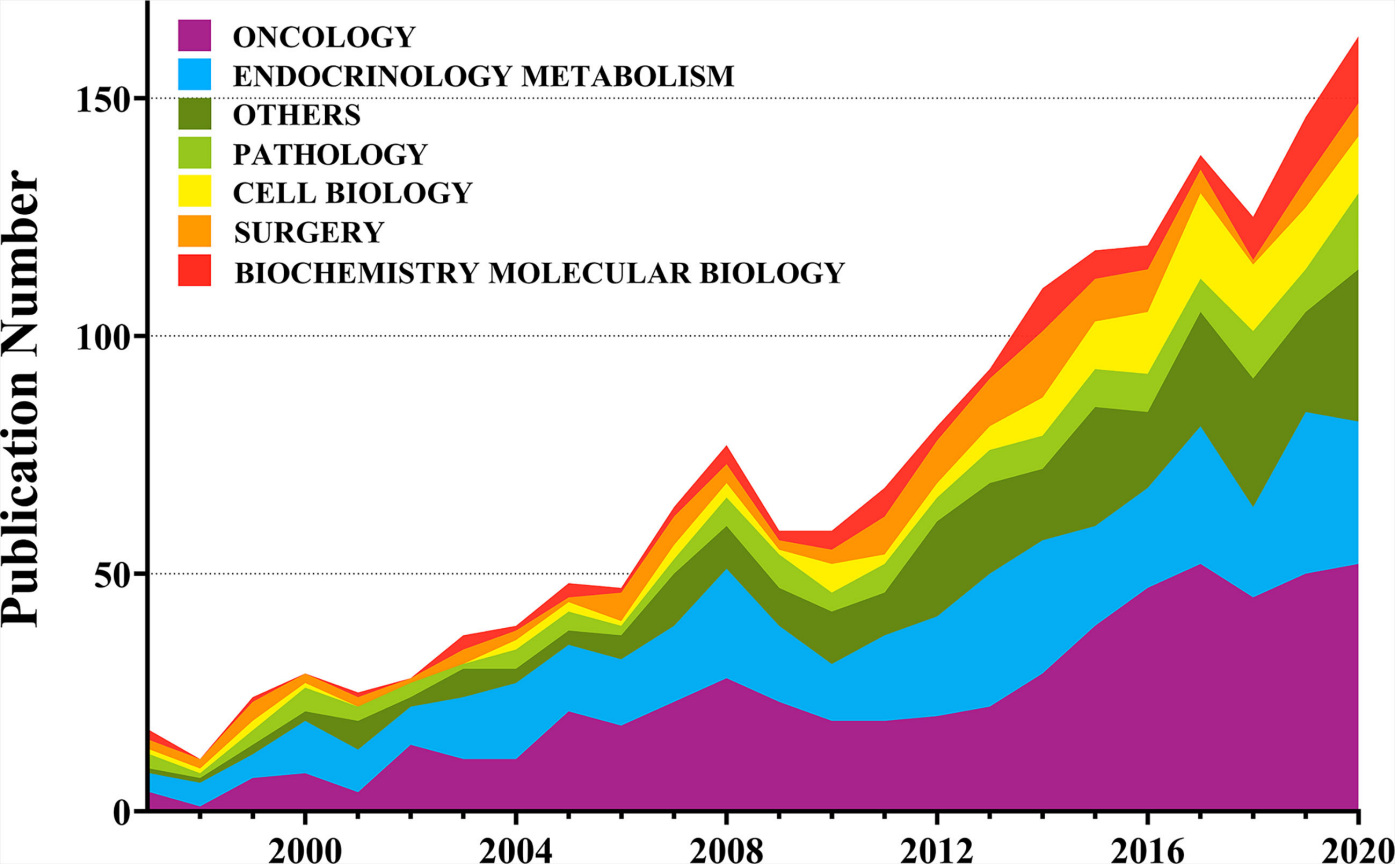
Quantity and growth trend of annual publications in ATC publications from 1997 to 2020.

From 1997 when the first identified publication appeared, the annual publication number of the ATC literature showed a progressive increase to the present (**Figure 2**). Over half of the literature belonged to the oncology and endocrinology, and metabolism cate-gory. Following categories were pathology, cell biology, surgery, and biochemistry molecular biology, etc. During these 24 years, the annual publications in pathology have been relatively stable, while these in cell and molecular biology have shown an upward trend.

### Leading Countries and Institutions

**Figure 3:**
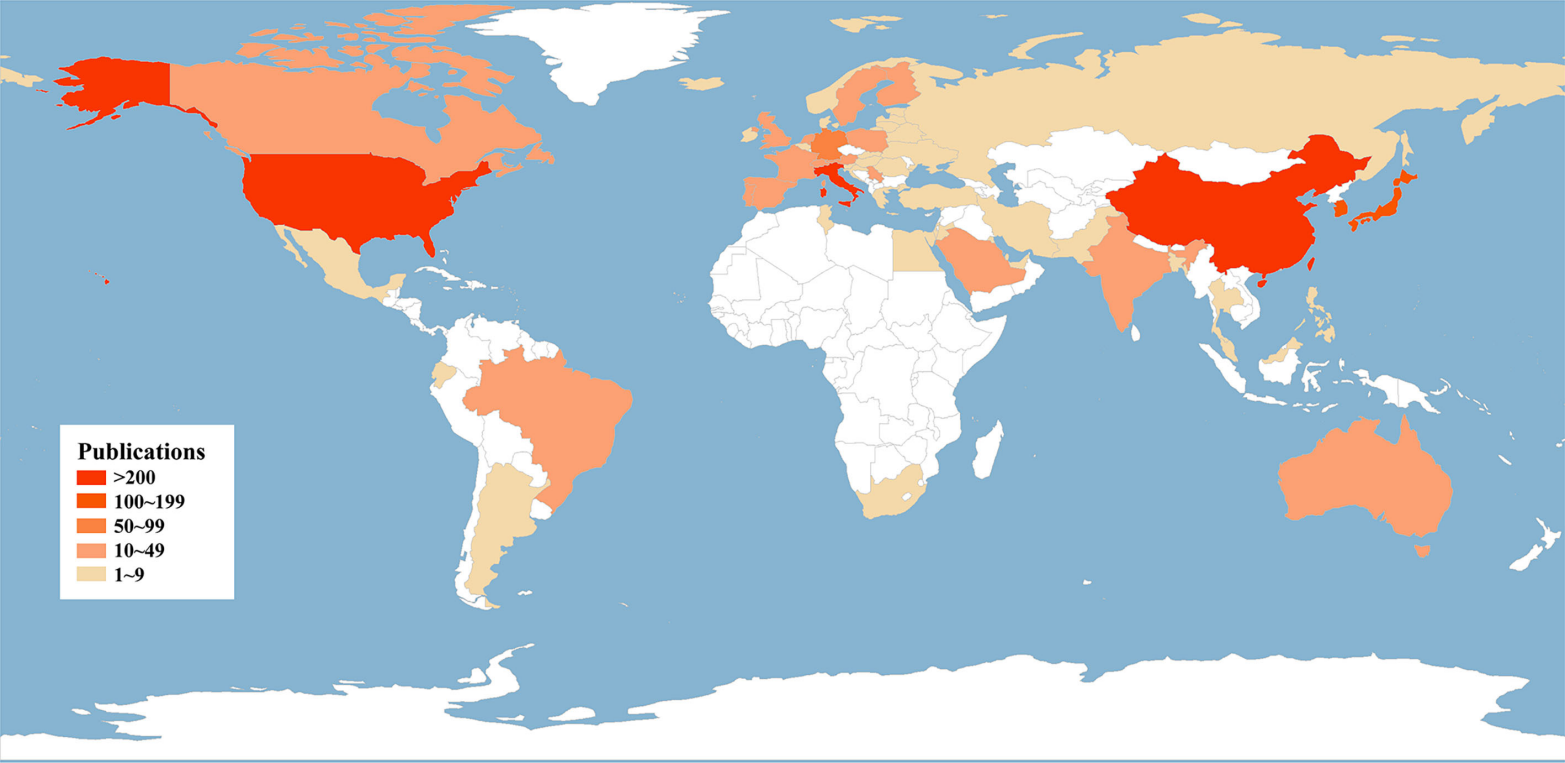
Global geographical distribution of ATC publications.

**Figure 4:**
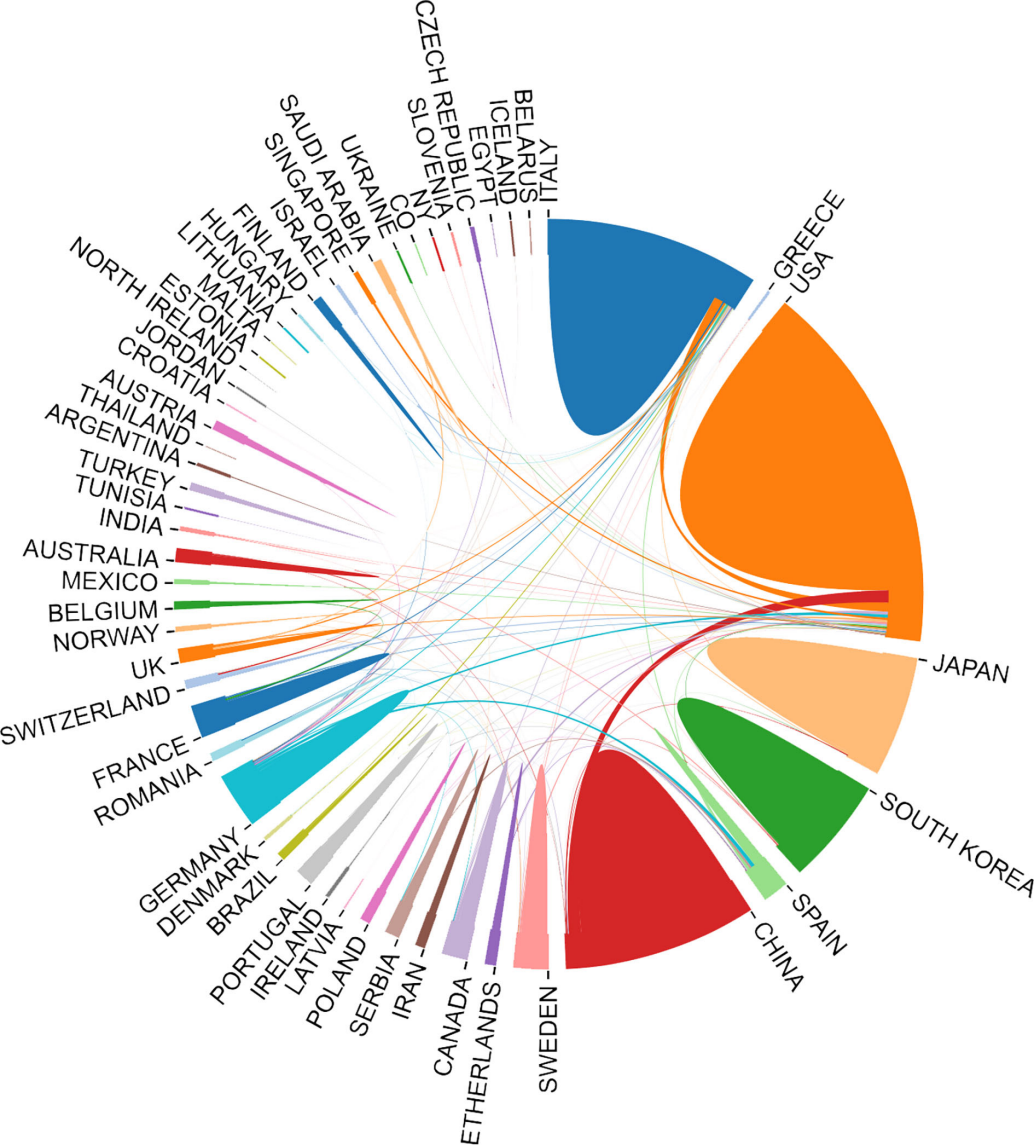
Cooperation pattern among contributed countries in ATC field.

**Figure 5:**
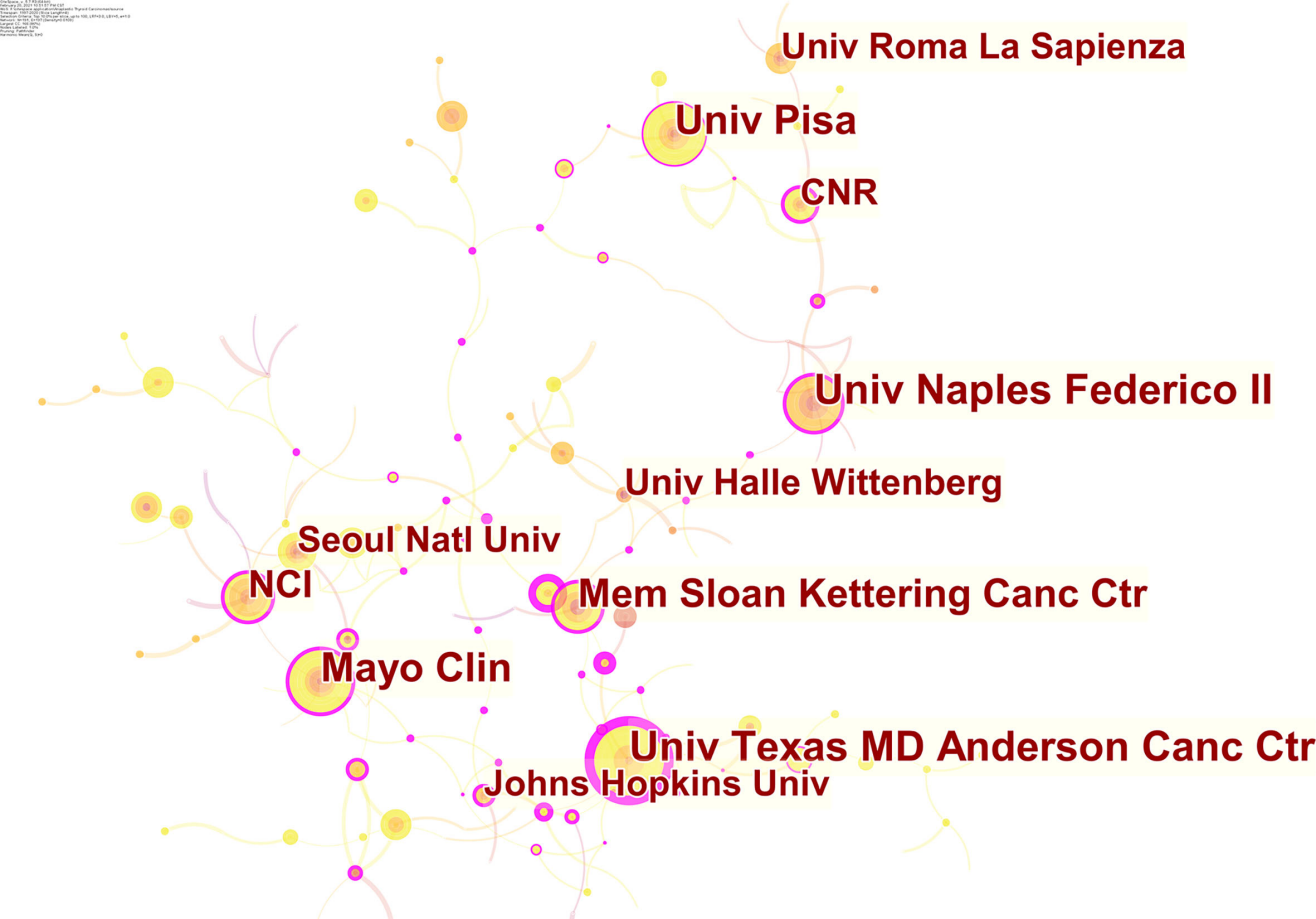
The network of contributed institutions in ATC field.

Contributed by over 64 countries and 1,460 institutions, a world map was drawn based on publication number (**Figure 3**). North America, East Asia, and Western Europe reached remarkable achievements. The United States participated in the maximum number of studies with the highest h-index of 71, followed by China and Italy (**Table 1**). Furthermore, intensive cooperation was observed among these countries (**Figure 4**). Distinguished from the leading institutions in other countries, Ito hospital from Tokyo was more committed to thyroid disorders than academic institutions founded by the government (**Table 1**). Benefited from the outputs of the MD Anderson Cancer Center, the University of Texas was honored as the most productive institution in the United States (**Table 1** and **Figure 5**).

**Table 1:** Top countries and institutions in ATC field.

Country	Articles	Citations	h-Index	Citations per article	Top country institution	Top Institution articles (%)
USA	486	19,860	71	40.86	UNIVERSITY OF TEXAS SYSTEM	87 (17.901%)
CHINA	263	3,629	30	13.80	SHANGHAI JIAO TONG UNIVERSITY	23 (8.745%)
ITALY	228	7,126	46	31.25	CONSIGLIO NAZIONALE DELLE RICERCHE	60 (26.316%)
JAPAN	159	3,993	34	25.11	ITO HOSP	25 (15.723%)
SOUTH KOREA	136	2,024	24	14.88	SEOUL NATIONAL UNIVERSITY	27 (19.853%)
GERMANY	90	3,398	30	37.76	MARTIN LUTHER UNIVERSITY HALLE WITTENBERG	29 (32.222%)
SWEDEN	43	1,605	21	37.33	KAROLINSKA INSTITUTET	19 (44.186%)
CANADA	42	1,293	17	30.79	UNIVERSITY OF TORONTO	19 (45.238%)
SPAIN	39	2,604	20	66.77	UNIVERSIDAD REY JUAN CARLOS	11 (28.205%)
FRANCE	35	1,962	18	56.06	UNICANCER	14 (40.000%)

### Most Active Journals and Highly Cited Publications

Academic journals serve as a medium for exchanging and disseminating knowledge among disciplines. Identified by WoS analysis system, more than 400 scholarly journals have published articles in the field of ATC. The 13 ‘core journals’ were detected according to Bradford’s law (**Table 2**): *Thyroid*, *Journal of Clinical Endocrinology & Metabolism*, *Endocrine-Related Cancer*, *Clinical Cancer Research*, *Oncotarget*, *Plos One*, *Cancer Research*, *Oncology Reports*, *Anticancer Research*, *Endocrine Pathology*, *Head and Neck-Journal for the Sciences and Specialties of the Head and Neck*, *Oncology Letters*, and *International Journal of Oncology* (27, 28). The impact factor, quartile, and categories were retrieved from JCR. Consistent with the former findings (**Figure 2**), most of the listed journals were categorized into “Oncology” and “endocrinology & metabolism” parts. Apart from *Oncotarget*, which has been de-listed from WoS in 2017, six out of the 12 journals were located in JCR quartile one and evaluated as high-quality scientific publications under the policy of the WoS evaluation system (**Table 2**).

**Table 2:** The core journals that published articles in ATC field.

Journal	Published numbers (%)	IF 2019	SJR 2019	JCR quartile	Categories
THYROID	114 (7.641%)	5.309	2.53	Q1	ENDOCRINOLOGY & METABOLISM
J CLIN ENDOCR METAB	94 (6.300%)	5.399	2.48	Q1	ENDOCRINOLOGY & METABOLISM
ENDOCR-RELAT CANCER	45 (3.016%)	4.800	1.55	Q1	ONCOLOGY; ENDOCRINOLOGY & METABOLISM
CLIN CANCER RES	36 (2.413%)	10.107	5.24	Q1	ONCOLOGY
ONCOTARGET	35 (2.346%)	NA	NA	NA	ONCOLOGY; CELL BIOLOGY
PLOS ONE	26 (1.743%)	2.740	1.02	Q2	MULTIDISCIPLINARY SCIENCES
CANCER RES	23 (1.542%)	9.727	4.05	Q1	ONCOLOGY
ONCOL REP	23 (1.542%)	3.417	0.97	Q2	ONCOLOGY
ANTICANCER RES	22 (1.475%)	1.994	0.72	Q4	ONCOLOGY
ENDOCR PATHOL	22 (1.475%)	3.168	0.89	Q2	ENDOCRINOLOGY & METABOLISM; PATHOLOGY
HEAD NECK-J SCI SPEC	20 (1.340%)	2.538	1.16	Q1	OTORHINOLARYNGOLOGY; SURGERY
ONCOL LETT	18 (1.206%)	2.311	0.67	Q3	ONCOLOGY
INT J ONCOL	17 (1.139%)	3.899	1.16	Q2	ONCOLOGY

SJR, SCImago Journal Rank. NA represents a missing value.

The highly cited and the most impact articles were also identified along with their characteristics (**Table 3**). Nine out of the ten articles were corresponded by the United States and one by Portugal, which was interrelated with the leading H-index of the United States (**Table 1**). Their published years ranged from 1999 to 2016, and all of them were located in JCR Q1. Among these highly cited science achievements, one out of the ten articles was the guideline for the management of ATC patients (29). Six papers were dedicated to genomics or genetic mutations, where B-type Raf kinase (*BRAF*), telomerase reverse transcriptase (*TERT*), etc., became the hot genes and the phosphatidylinositol 3-kinase/Akt pathway was the focused pathway (18, 30–34). It remains a challenge for the effective cure of ATC, among which targeted therapy has been regarded as the current therapeutic orientation. Three out of the ten articles were clinical studies of targeted therapy drugs, including vemurafenib for *BRAF^V600E^* mutations, combretastatin A-4 phosphate (CA4P) for vascular disruption, and pazopanib for tyrosine-kinase inhibition (35–37). These drugs were tested for efficacy not only in ATC but also in a variety of cancers.

**Table 3:** The characteristics of highly cited and the most impact classic articles in ATC field.

Rank	Total citations	Article title	Journal	Published year	Country	IF 2019
1	881	Vemurafenib in Multiple Nonmelanoma Cancers with BRAF V600 Mutations	NEJM	2015	USA	74.699
2	378	Genomic and transcriptomic hallmarks of poorly differentiated and anaplastic thyroid cancers	J CLIN INVEST	2016	USA	11.864
3	371	American Thyroid Association Guidelines for Management of Patients with Anaplastic Thyroid Cancer	THYROID	2012	USA	5.309
4	346	A phase I pharmacokinetic and translational study of the novel vascular targeting agent combretastatin A-4 phosphate on a single-dose intravenous schedule in patients with advanced cancer	CANCER RES	2002	USA	9.727
5	339	Highly prevalent TERT promoter mutations in aggressive thyroid cancers	ENDOCR-RELAT CANCER	2013	USA	4.800
6	333	Mutational Profile of Advanced Primary and Metastatic Radioactive Iodine-Refractory Thyroid Cancers Reveals Distinct Pathogenetic Roles for BRAF, PIK3CA, and AKT1	CANCER RES	2009	USA	9.727
7	287	Efficacy of pazopanib in progressive, radioiodine-refractory, metastatic differentiated thyroid cancers: results of a phase 2 consortium study	LANCET ONCOL	2010	USA	33.752
8	278	Genetic alterations and their relationship in the phosphatidylinositol 3-kinase/Akt pathway in thyroid cancer	CLIN CANCER RES	2007	USA	10.107
9	273	TERT Promoter Mutations Are a Major Indicator of Poor Outcome in Differentiated Thyroid Carcinomas	J CLIN ENDOCR METAB	2014	Portugal	5.399
10	266	Frequent mutation and nuclear localization of beta-catenin in anaplastic thyroid carcinoma	CANCER RES	1999	USA	9.727

### Development Skeleton and Scientific Landscapes

**Figure 6:**
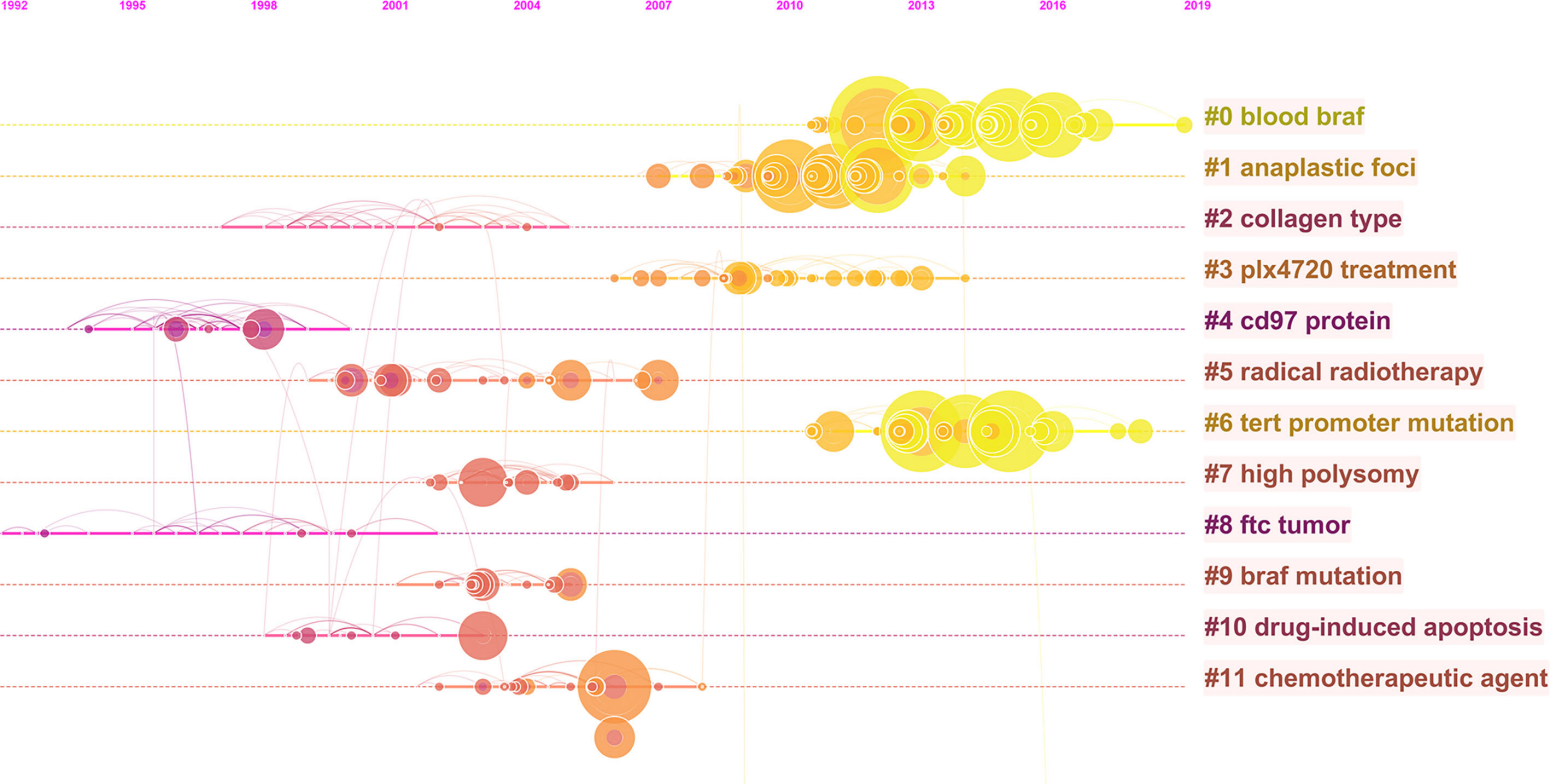
The co-citation timeline map of references in ATC field from 1997 to 2020. Co-cited references that are repeatedly cited in ATC literature were clustered and identified by CiteSpace. Nodes on the map represented referenced documents. The trend of co-coted literature subjects reflected the shift of research concerns. The years were arranged horizontally at the top, and the label of each cluster was displayed at the end of the cluster’s timeline.

Co-citation analysis is a universal technique to characterize the internal structure of intellectual knowledge in terms of co-cited references (38). Through clustering the co-cited documents, an insight of the topology of documents was provided. Therefore, we performed co-citation analysis of ATC to explore its historical developments and scientific landscapes. As a result, 12 main clusters were generated and labeled by phrase extracted from the abstracts (**Figure 6**). The map can be approximately divided into three periods: early stage, middle stage, and current stage. The earlier studies were mainly interested in collagen type (#2), CD97 protein (#4), and FTC tumor (#8). The current stage cared more about blood *BRAF* (#0), anaplastic foci (#1), plx4720 treatment (#3), and *TERT* promoter mutation (#6). Concerns on radical radiotherapy (#5), high polysomy (#7), *BRAF* mutation (#9), drug-induced apoptosis (#10), and chemotherapeutic agent (#11) appeared in the middle stage, acting as a bridge between the early stage and the current studies.

**Figure 7:**
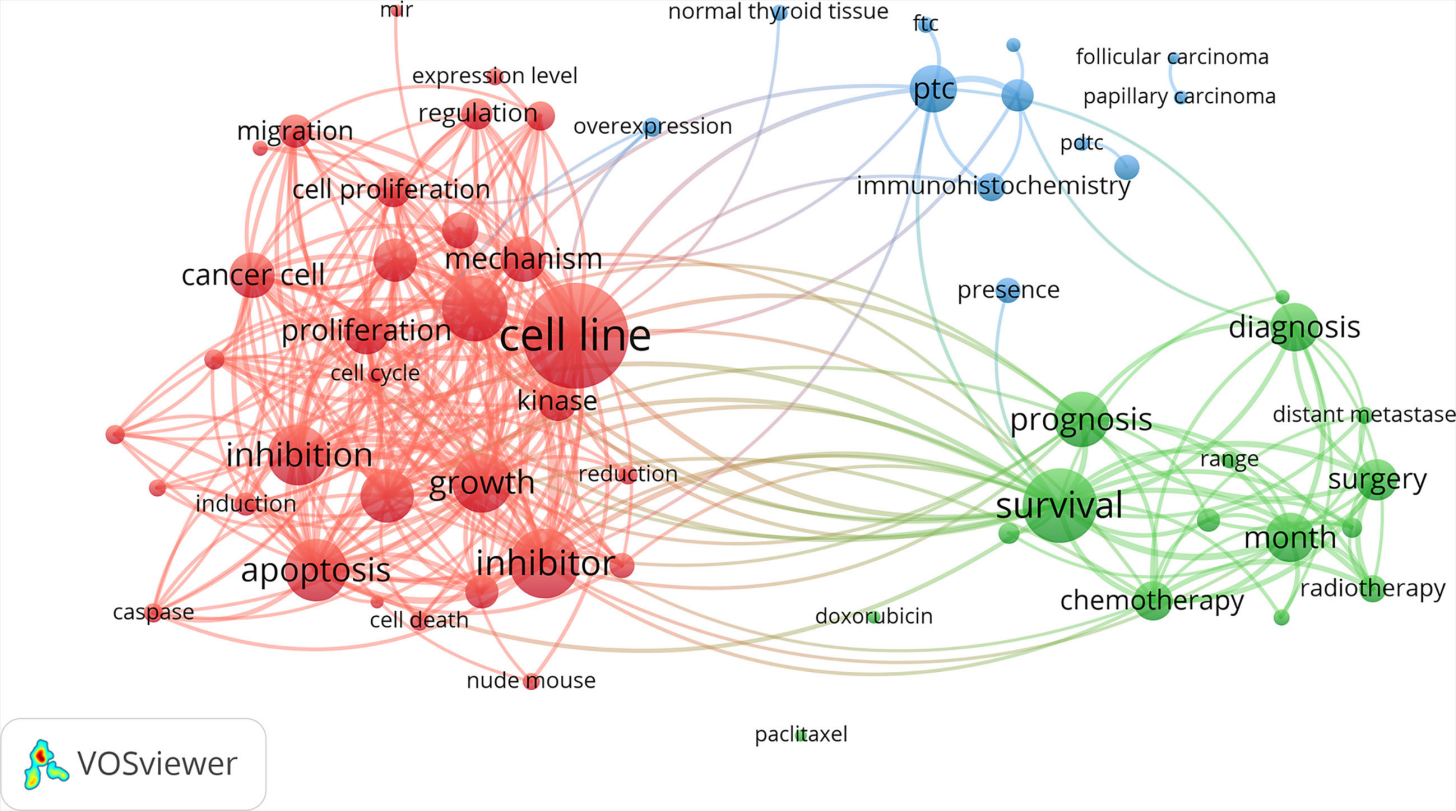
Clustering network of terms in ATC publications from 1997 to 2020. The most common terms used in the scientific literature were investigated to identify relationships among the extracted terms. Terms were automatically exacted from titles and abstracts and divided into clusters by natural language processing techniques of VOSviewer.

After excluding units of repeated and irrelevant items, we used natural language processing techniques by VOSViewer software to identify and cluster term phases to outline the scientific landscapes (**Figure 7**). The identified terms were automatically divided into three clusters. The red cluster in the left contained the most terms that related to experimental medicine. It enriched cell proliferation, growth, regulation, migration, apoptosis, death and other terms related to cell life process. Specific concerns, such as cell cycle, caspase, kinase, etc., were also highlighted. The green cluster in right was where terms about clinical investigation predominated. There were terms related to the diagnosis, treatment and prognosis of ATC concentrated in the green cluster. For instance, various therapy containing chemotherapy, surgery and radiotherapy, were gathered. The interrelated thyroid diseases including FTC and PTC were gathered in the blue cluster at the top of the map.

### Focus Shift and Research Frontiers

**Figure 8:**
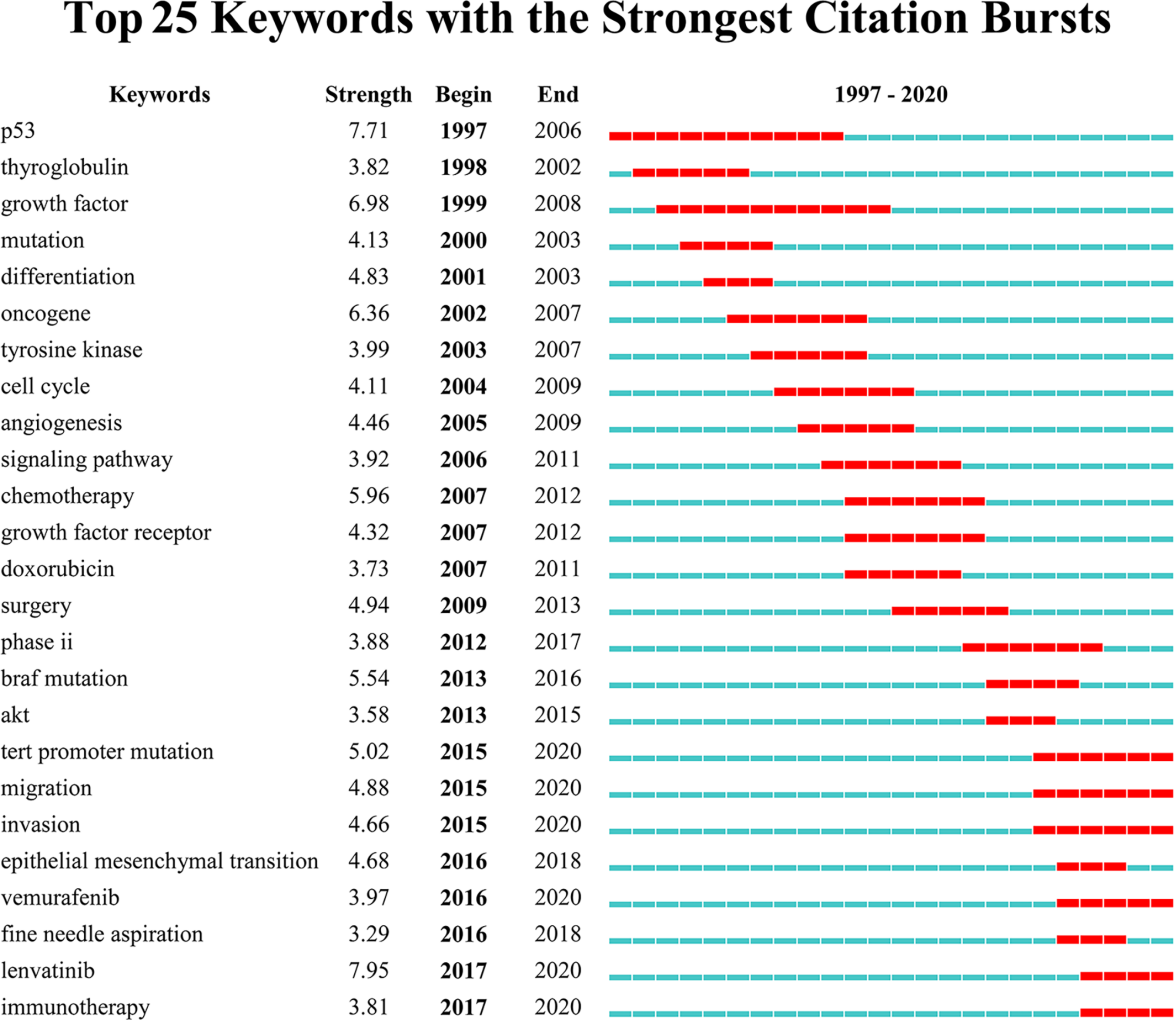
Top 25 keywords with the strongest citation bursts. Keywords with the strongest citation bursts in the scientific literature were analyzed and visualized in the keyword’s bursts map. Each short line represented a year and lines in red stood for the burst detection years. Keywords with red lines extending to the latest year can indicate the research frontiers in a short period of time in the future.

Keywords with intense bursts in a short period can act as a sensitive indicator to reflect the research focus. Recent burst keywords provide researchers the possible research frontiers in a short future. A keyword burst map was generated by CiteSpace, where the strength and the beginning or ending year of burst was shown (**Figure 8**). The strength reveals the burst intensity, and the burst year indicates the transformation of the research focus and its duration. Progress has been made in the genomics and genetics of ATC. Some nonspecific biomarkers, such as thyroglobulin (begin in 1998) and growth factor (begin in 1999), have been reported in the early period. Afterwards, P53 (begin in 1997), mutation (begin in 2000), oncogene (begin in 2002), *BRAF* mutation (begin in 2013), and *TERT* promoter mutation (begin in 2015) generalized the research focus transformation over the past 24 years. The searching for possible mechanisms and pathways keeps moving where tyrosine kinase (begin in 2003), cell cycle (begin in 2004), angiogenesis (begin in 2005), Akt (begin in 2013), and epithelial–mesenchymal transition (begin in 2016) provided clues for researchers. Fine needle aspiration (begin in 2016) has been performed as a new minimally invasive diagnostic technique in recent years. The treatment also transferred with time where chemotherapy (begin in 2007) and surgery (begin in 2009) burst successively but neither of them continued to the present. Trials on potential drugs focused on doxorubicin (begin in 2007) at the beginning of the 21st century, and then transferred to vemurafenib (begin in 2016) and lenvatinib (begin in 2017) in the recent researches. What’s more, immunotherapy (begin in 2017) appeared recently and kept bursting till 2020.

## Discussion

Although rare, ATC is one of the most aggressive and lethal human tumors. ATC studies make great sense for patients to reduce mortality and their financial burden. As far as we know, this study was the first systematic bibliometric assessment of the scientific publications about ATC from 1997 to 2020. Previous thyroid-related bibliometric studies paid attention to hypothyroidism in pregnancy, Bisphenol A and thyroid hormones, or picked one-hundred most-cited articles focused on thyroid research (39–41). We aimed to obtain an overview from two perspectives to outline the ATC research field. One is the general characteristics, including publication numbers, contributed countries, core journals, etc. Another is the substance contents, including developmental skeletons, research frontiers, etc.

The number of annual publications in the field of ATC has kept a fluctuant growth during the past twenty years. Flourishing in publications and researches will promote the development of the ATC field. It is noteworthy that the annual number of publications on the Cell Biology or Biochemistry Molecular Biology categories was increasing, which suggested a deeper understanding of the ATC’s pathogenesis (**Figure 2**). The analysis of leading countries and institutions came to a result that the United States, China, Italy, Japan, and South Korea were listed as the top five contributed countries (**Table 1** and **Figure 3**). This global distribution of ATC publications corresponded to the prevalence of thyroid cancers and the level of development in different countries (42). From the perspective of international cooperation, the United States cooperated with China and Italy frequently while Japan and South Korea had little cooperation with other countries (**Figure 4**). It is suggested that global cooperation be further strengthened. As one of the largest cancer centers in the world, MD Anderson Cancer Center has been keeping active academic research. Other influential institutes included Mayo Clinic in Rochester, University of Naples Federico II in Naples, and the University of Pisa in Pisa (**Figure 5**).

In the early stage of co-citation analysis, active citations on FTC tumors, collagen types, and CD97 protein were identified (**Figure 6**). As a subcategory of thyroid cancer, ATC was frequently explored and compared with FTC and other thyroid cancers in the aspect of prevalence, pathogenesis, and diagnosis (43–45). Meanwhile, the unique characteristics of ATC were gradually being explored. For instance, its aggressive characteristics have been suspected to be closely related to the alternation of tumor stroma, such as collagen. Its non-differentiation was likely to perform abundant expression of dedifferentiation markers, such as CD97 (46–49). Later citations noted the high polysomy phenomenon, *BRAF* mutation, and *TERT* promoter mutation in ATC patients. These profound insights into the genetics of ATC have emerged due to the breakthrough of biotechnology and nucleic acid sequencing technologies. It was reported that *BRAF* ^V600E^ mutation was commonly associated with ATCs. Specifically, about 40% of ATCs patients have been detected with *BRAF* ^V600E^ mutations (17, 18). As a common alteration in ATC, its positive detection is conductive to the diagnosis of ATC patient, even though *BRAF* ^V600E^ mutation can be found in other tumor types (11, 13, 35, 50). Moreover, because ATC are such rare disease that patients are unlikely to purchase targeted drugs only for ATC, *BRAF*-targeted drugs approved for other common diseases imply an important clinical value in ATC treatment (11, 13, 35, 50).Recent citations noted anaplastic foci and blood *BRAF*. Compared to traditional traumatic diagnostic methods to recognize anaplastic foci, circulating cell-free DNA (cfDNA) in peripheral blood has become a potential candidate as biomarker in early tumor detection for its convenience and non-invasion (51, 52). It has been clinically found to possess high concordance rates in gene mutation identification between cfDNA analysis and tumor tissue sequencing (53). On the other hand, the citation of the articles related to chemotherapy and radiotherapy illuminated researchers were making great efforts in ATC’s therapy (54). However, ATC did not respond well to these standard therapies and the survival rate did not improve significantly (55). The advancement in the 21st century has brought a new class of chemotherapy, namely targeted therapy, which works against particular molecules that contribute to cells out of control. As mentioned above, several targeted drugs against *BRAF* (i.e., vemurafenib, etc.) have been developed and under clinical trials in various tumor types. Monotherapy with a BRAF inhibitor may result in initial clinical benefits, but this is for a short duration as acquired resistance is common after several-month treatment and leads to a significant relapse in almost all cases, including ATC (35, 56–58). It reminds us that ATC is more than simple *BRAF* mutant. If promising, consideration into the exploration of its resistance mechanisms should be taken as to develop dual inhibition targeted drugs. Moreover, looking for other mutant genes and abnormal pathways for appropriate targeted monotherapy or combination therapy will also be the point of the future work.

Discovered by the terms analysis system of VOSviewer, a landscape of several research topics was presented (**Figure 7**). In experimental medicine, topics on the mechanism of the growth, migration, apoptosis, inhibition, and death of cancer cells were noted. For the work on revealing the ATC mechanism, there are obstacles to overcome but some real achievements that have been made. It has indicated that some mutation events (i.e., *BRAF*, mitogen-activated protein kinase pathway, etc.) initiate thyroid cancer development and predispose to tumor dedifferentiation while several key nodes (i.e., *TERT*, *TP53*, *PI3K*-*AKT*-mTOR pathway, etc.) altered promote tumor progression and contribute to tumor aggressivity. The persistence of *BRAF* mutations throughout thyroid cancer development and the acquisition of additional mutations in ATC indicate a stepwise accumulation of mutations, which holds out the hypothesis of tumor progression from preexisting well-differentiated thyroid cancer to ATC (1, 11, 59). Besides, the bioinformatic analysis found that genes related to the cell cycle (i.e., *CDKN2A*, *CDKN2B*, *CCNE1*, etc.) were mutated or abnormally expressed in ATC, which might provide another thought in advanced thyroid cancers (17, 60). Except for encoding genes, non-coding genes have emerged as critical regulators in ATC, but its alteration of expression profile and dysfunction need to be further confirmed yet (61–63). In clinical research, diagnosis, therapeutics (i.e., chemotherapy, surgery, radiotherapy, etc.), and prognosis (i.e., survival, range, distant metastases, month, etc.) were the major topics. Great efforts have been made in these subfields, but there is little progress in the early-stage diagnosis and effective treatment of ATC yet, leading to extremely poor prognosis with 5-month median survival (11, 16). When possible, surgery is generally the critical step in treatment of anaplastic thyroid cancer. Patients with ATC cells only inside the thyroid gland (stage IVA) or outside the thyroid capsule involving locoregional lymph nodes (stage IVB) but without distant metastasis (stage IVC), if R0/R1 resection can be achieved, surgery should be considered for its survival benefits and prevention of future complication. Additionally, surgical resection combined with postoperative radiotherapy and/or chemotherapy would conduce to kill the residual tumor confer a longer survival (11). However, the fact that most of the patients present at diagnosis are in a state of extensive extrathyroidal tumor diffusion, which make complete resection unlikely. At this time, radiotherapy and/or chemotherapy represent the standard treatment of choice (16, 29). Therapies taken into account do not involve radioactive iodine (RAI) for its invalidation in ATC, because ATC cells are so dedifferentiated to lack the ability of iodine uptake from the blood (64). Whether traditional therapy improves patient outcome is still unclear (65). ATC patients might obtain little benefit from traditional therapy but bear more negative effects. Mutation-guided targeted therapeutic strategies might be a candidate opportunity for ATC patients, especially in advanced or initially unresectable ATC. Through the interference against central mutant molecular system involved in the development of cancer, it blocks tumor cells with precision and efficiency, which might work when traditional therapy does not work or might work in concert with traditional therapy to enhance its efficacy. Mutation-guided targeted therapies are currently the focus of anticancer drug development (11, 50, 65, 66).

Unlike terms, keywords are supplied by authors, generalizing the theme and features of the article. Burst keywords reveal the research hotspots and their transformation from clinicopathology to genetics and advanced therapies. Particularly, burst keywords that continue to the present indicate the potential trends and possible frontiers in the field of ATC (**Figure 8**). The latest burst keywords include “*TERT* promoter mutation”, “migration”, “invasion”, “vemurafenib”, “lenvatinib”, and “immunotherapy”. Initially, some classical oncogenes, such as *TP53* that often occurred in other malignant tumors, were observed with dysfunction in ATC patients (67). Besides, some critical points in oncology, including the cell cycle regulation and receptor tyrosine kinases (RTKs, the receptors for a wide array of growth factors like EGFR, VEGF, RET, etc.), were detected to be in a state of disorder (17, 60, 68–70). The early studies relied on a set of well-known cancer-associated genes (i.e., *BRAF*, *TP53*, *RAS*, etc.) and a general consensus on the characteristics of cancer cells, but whether these alterations are dominant in ATC or as subsequent phenomenon needs to be further confirmed. What’s more, these targeted gene detections and feature observations are likely to omit quite a few unknown critical genes and phenotypes (71). Later, with less costly and more efficient large-scale sequencing technology, more comprehension has been made in the characteristics of ATC. Besides *BRAF^V600E^*, which has been thoroughly discussed above, the discovery of the *TERT* promoter mutation in thyroid cancer was an important event, and much progress has occurred since then (30). Consistent with our previous results (**Figure 6**), studies of *TERT* gene are in an active stage. Telomerase reverse transcriptase (*TERT*) is a subunit component of telomerase with reverse transcriptase activity and participates in maintaining telomere ends and chromosomal stability, which is a critical member in tumor suppressive mechanism. Therefore, the abnormal activation of *TERT* might contribute to neoplastic progression (72). It was reported that ATC patients possessed a higher *TERT* promoter mutation burden with 73% prevalence (18, 73). *TERT* promoter mutations represent a prominent new oncogene in ATC and this advance in the comprehension of ATC pathogenesis pattern has facilitated a promising new development in diagnosis and treatments. In the era of individualized and precision medicine, molecular targeted therapy and immunotherapy develop quickly (74). Recently a number of recent advances in targeted therapies hold out if not the hope of a cure, then at least the possibility of partial responses to postpone ATC progress (65). Several novel agents have been evaluated in ongoing clinical trials among ATC patients, which are roughly divided into two categories—targeting genetic aberrations and targeting the tumor microenvironment (75). Taking the *BRAF* targeted therapy mentioned above as an example, although it has been reported the tendency to drug resistance of vemurafenib in ATC, *BRAF* inhibitors have been still placed great expectations to be leveraged into ATC as a targeting genetic aberrations drug. Recent studies have attempted into the abnormal downstream pathways centered on BRAF, especially the most downstream pathway component, to overcome upstream resistance (71). Novel *BRAF* inhibitors (i.e., dabrafenib, etc.) in combination with downstream pathways (i.e., MEK inhibitor trametinib, etc.) showed a significant response rate (RR) in ATC patients with the *BRAF* ^V600E^ mutation. This combination is now approved by the FDA (50). *TERT* promoter mutation represents an attractive target in ATC but telomerase inhibitors have not demonstrated their clinical efficacy yet (76). It is recommended to consider an appropriate personalized targeted therapy according to mutants present, if possible. Furthermore, several mechanisms that favor tumor growth and dissemination (e.g., angiogenesis, invasiveness, and metastasis) are promising alternatives against biological behavior and microenvironment of tumor and sever an adjuvant therapy to delay ATC progression. The interest has been rosed in a series of tyrosine kinase inhibitors which target against VEGFR, EGFR, FGFR, RET and so on. Nevertheless, only one multikinase inhibitor, lenvatinib, shows potentiality in clinical application yet (11, 68). In addition, several strategies of immunotherapy are attempting as novel types of ATC treatment (77). It has shown that ICAM-1 CAR T cells mediated ATC tumor killing were of validation *in vitro* and in animal models (78). The induction of immune checkpoint inhibitors, including anti-programmed cell death protein 1/programmed cell death ligand 1 (PD-1/PD-L1), have shown promising results in mice with systemic ATC (79). However, their potential efficacy still lacks sufficient experimental and clinical data, which needs to undergo more research verification. Studies on targeted therapy, immunotherapy, and systematic combination therapy might indicate the frontier of the ATC field.

## Conclusions

To outline the landscapes of ATC research, we constructed several scientific visualized maps. Growth trends, leading countries and their collaborations, core journals, co-citation analysis, term clustering, and burst keywords were explored to give a comprehensive bibliometric overview. Since ATC is a type of malignancy with a horrible lethal rate, researchers have made numerous efforts to figure out its reprogramming of gene expression and key mechanism in driving its dedifferentiation, invasion, and migration process. Genetic testing is becoming widespread and accessible in clinical diagnosis of ATC, and its outcome could point out possible drug targets. Many therapeutic modes were visualized in the timeline, some of which are gradually being replaced by more advanced and precise treatments. Studies on targeted therapy against genetic aberrations or tumor microenvironment, immunotherapy and systematic combination may lead the future academic directions, which deserve more resource investments and financial supports.

## Data Availability Statement

Publicly available datasets were analyzed in this study. This data can be found here: http://apps.webofknowledge.com.

## Author Contributions

This work was conceived by HS. Data was collected and downloaded by YY. HW and KW helped to check and verify data as two independent investigators. The visualization work was performed by KW. The manuscript was written by HW and YY. HS helped to revise manuscript and proposed constructive opinions. All authors contributed to the article and approved the submitted version.

## Conflict of Interest

The authors declare that the research was conducted in the absence of any commercial or financial relationships that could be construed as a potential conflict of interest.

## Publisher’s Note

All claims expressed in this article are solely those of the authors and do not necessarily represent those of their affiliated organizations, or those of the publisher, the editors and the reviewers. Any product that may be evaluated in this article, or claim that may be made by its manufacturer, is not guaranteed or endorsed by the publisher.
